# Differentially expressed miRNAs offer new perspective into cave adaptation of *Astyanax mexicanus*

**DOI:** 10.1111/nyas.15300

**Published:** 2025-03-13

**Authors:** Tathagata Biswas, Huzaifa Hassan, Nicolas Rohner

**Affiliations:** ^1^ Stowers Institute for Medical Research Kansas City Missouri USA; ^2^ Institute for Integrative Cell Biology and Physiology University of Münster Münster Germany

**Keywords:** *Astyanax*, cavefish, development, gene expression regulation, miRNA

## Abstract

*Astyanax mexicanus*, a species with both surface‐dwelling and multiple cave‐dwelling populations, offers a unique opportunity to study repeated adaptation to dark and resource‐scarce environments. While previous work has identified large‐scale gene expression changes between morphs under even identical laboratory conditions, the regulatory basis of these expression differences remains largely unexplored. In this study, we focus on microRNAs (miRNAs) as key regulators of gene expression. Our analysis identified 683 mature miRNAs, establishing the first comprehensive catalog of miRNAs for this species. We identified a unique subset of differentially expressed miRNAs common to all studied cave‐dwelling populations, potentially orchestrating the nuanced gene expression patterns required for survival in the cave milieu. Furthermore, we performed in silico target prediction of these miRNAs, revealing possible roles in developmental and metabolic pathways pivotal for thriving in nutrient‐limited cave conditions. Interestingly, we also observed that Molino, which is the “youngest” of the three cavefish analyzed in this study, exhibited the most abundant number of differentially expressed mature miRNAs among the cave morphs. The comprehensive miRNA catalog generated, along with the insight into their differential expression across different morphs, will guide future investigations into the intricate world of miRNA‐mediated evolution of complex traits.

## Graphical abstract

*Astyanax mexicanus*, featuring surface and cave populations, offers a unique model to study adaptations to dark, resource‐scarce environments. Our study focuses on miRNAs as regulators of gene expression in cavefish adaptation. We identified 683 mature miRNAs, uncovering the possibility of miRNA‐mediated mechanisms crucial for cave survival, including developmental, metabolic, circadian rhythm, and oxidative stress pathways. This comprehensive catalog will aid future studies on miRNA‐mediated evolution.

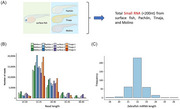

## INTRODUCTION

The fish species *Astyanax mexicanus* is an exceptional animal model for understanding adaptation to dark and nutrient‐limited environments. This species exists as river‐dwelling surface fish and cave‐dwelling populations that remain fertile with each other and can be kept in the laboratory. Less than 200,000 years ago, the ancestral surface fish colonized multiple caves in the Sierra de El Abra and neighboring mountain regions of Mexico through multiple independent events.^1^, ^2^ Inside each of these caves, under similar environmental pressures, *Astyanax* evolved a range of similar traits that enabled the cave morphs to not only survive but thrive in the dark and, therefore, biodiverse and nutrient‐limited cave environment.^3^, ^4^, ^5^, ^6^, ^7^, ^8^, ^9^ As a consequence, the cave morphs show significant and heritable changes to their transcriptomic landscape when compared to the extant surface fish.^5^, ^10^, ^11^, ^12^, ^13^ While the role of cis‐regulatory enhancers in these transcription changes has been studied,^13^ the exploration of other mechanisms of transcriptional regulation remains less understood.

One particularly important mechanism that warrants further investigation is the role of noncoding RNAs in modulating gene expression in *A. mexicanus*. A class of noncoding RNA that plays an important role in shaping gene expression profiles are microRNAs (miRNAs). miRNAs are a class of small endogenous noncoding RNA molecules, typically averaging around 18–25 nucleotides in length, which act post‐transcriptionally to regulate gene expression.^14^, ^15^, ^16^, ^17^ Exported into the cytoplasm from the nucleus as short hairpin structures, one of the strands of a mature miRNA is eventually loaded into the RNA‐induced silencing complex. The silencing complex most frequently targets the 3′ UTR region of mRNAs through complementary base pairing and fine‐tunes gene expression through translational repression or mRNA degradation.^18^, ^19^, ^20^ A single miRNA can potentially target multiple mRNAs, and conversely, a single mRNA can be a target of many different miRNAs. Although this suggests a complex and intertwined regulatory potential for miRNAs, in general, as demonstrated by multiple experimental studies, a given miRNA primarily targets a single or a limited number of mRNAs to drive specific phenotypic outcomes.^21^, ^22^, ^23^, ^24^, ^25^, ^26^ With the potential to alter the transcriptomic profile and drive specific phenotypes, miRNAs have been extensively studied in multiple animal models, including vertebrates and invertebrates.^27^, ^28^, ^29^

For ray‐finned fish, there exists an extensive catalog of miRNAs in the form of the FishmiRNA database, which details miRNA annotation and expression for 12 different species, including 10 teleost and two holosteans.^30^ The database provides comprehensive insight into the number of mature miRNAs, miRNA genes, and genomic locations associated with each miRNA in these 12 species. While zebrafish have the highest number of mature miRNAs among the listed fish species, with a count of 494, the fish shortfin molly exhibited the lowest number, with 328 mature miRNAs. The mature miRNAs in zebrafish originated from 385 different miRNA gene locations, of which 308 have orthologs that could be found in other species. Altogether, the most recent updated version of the FishmiRNA database lists a total of 4787 mature miRNAs across 12 species of ray‐finned fish. Likewise, the miRBase database boasts a collection of miRNAs from 271 distinct organisms.^31^, ^32^ However, despite having been studied extensively in other fishes and organisms, miRNA's potential role in altering the transcriptomic profile of cavefish, and its possible role in its development and evolution, remains unexplored.

In this study, we focused on 24‐h‐post‐fertilization (hpf) embryos to isolate and annotate mature miRNAs expressed in four different morphs of *A. mexicanus*: surface fish, and three independently or parallelly evolved populations of cavefish (Pachón, Tinaja, and Molino). Using the miRDeep2 package^33^ to call for novel miRNAs in *A. mexicanus*, we identified as many as 683 different mature miRNAs. Our comparative analysis of miRNA expression between three cave‐dwelling and surface‐dwelling populations of *A. mexicanus* identified a cave‐specific set of differentially expressed miRNAs and their putative 3′ UTR targets. Gene Ontology (GO) analysis of these targets suggests that miRNA regulation may play a critical role in cave adaptation by influencing developmental and metabolic pathways. This study not only establishes the miRNA catalog for this species but also underscores the potential role of miRNA‐mediated regulation in the evolutionary response to extreme environments.

## MATERIALS AND METHODS

### Small RNA isolation and sequencing

We collected 50 embryos of each morph of *A. mexicanus* at the 24‐hpf developmental stage. Surface and Pachón develop comparably until 24 h,^34^ and with Tinaja and Molino, attention was paid to make sure the stages of the collected embryos were all comparable and that the developmental differences in timing and allometry had a negligible effect upon subsequent analysis. After a 1x PBS wash, samples were flash frozen and homogenized. The small RNA population from each morph was isolated using the mirVana miRNA Isolation Kit (ThermoFisher Catalog number: AM1560) following the manufacturer's instructions.

Small RNA‐seq libraries were generated from 50 ng of enriched small RNA, as assessed using the Bioanalyzer (Agilent). Libraries were made according to the manufacturer's directions for the TruSeq Small RNA Library Prep Kit (Illumina, Cat. No. RS‐200‐0012). The resulting libraries were size selected for 145–160 bp fragments using Novex 6% TBE gels (Invitrogen, Cat. No. EC6265BOX) to retain only the small RNA inserts. Size‐selected libraries were checked for quality and quantity using the Bioanalyzer (Agilent) and Qubit Fluorometer (Life Technologies). Libraries were pooled, requantified, and sequenced as a 75‐bp single read on an Illumina NextSeq 500 high‐output flow cell utilizing NextSeq Control Software 2.2.0.4. Following sequencing, Illumina Primary Analysis version RTA 2.4.11 and bcl2fastq2 v2.20 were run to demultiplex reads for all libraries and generate FASTQ files.

### Adapter removal and filtering of reads

The TGGAATTCTCGGGTGCCAAGG adapter was removed for all samples of small RNA‐seq using Cutadapt^35^ (v2.5). A histogram showing the total sequencing range is depicted in Figure S1. Reads without an adapter and shorter than 18 bp were discarded. We excluded reads aligning to rRNAs, scaRNAs, snoRNAs, and snRNAs (Table S1). Additionally, reads aligning to tRNAs were removed (Table S2). As annotated tRNAs for *A. mexicanus* 2.0 are unavailable, we used tRNAscan‐SE^36^ (v2.0.6) to search for *A. mexicanus* 2.0 tRNAs using *Danio rerio* (GRCz11) tRNAs from GtRNAdb (the genomic tRNA database).

### Novel miRNA identification

The reads remaining after filtering out rRNAs, scaRNAs, snoRNAs, snRNAs, and tRNAs from all samples were concatenated and the miRDeep2^33^ (v3) tool was used to identify novel miRNAs. First, the mapper.pl module was used to align the reads to the *A. mexicanus* 2.0 genome from NCBI (Assembly GCF_000372685.2). Next, the miRDeep2.pl module was run on the mapped arf file using the reference genome and known mature *D. rerio* miRNAs from miRBase (v22) as supporting miRNAs from related species.

The resulting novel miRNAs were further filtered based on the following criteria: mirDeep2 score ≥10, mature read count ≥10, and a significant randfold *p*‐value. Novel miRNAs with a matching seed sequence to *D. rerio* miRNAs from miRBase were retained, even if they did not meet the above criteria. The CD‐HIT^37^ (v4.6) tool was used to collapse the redundant novel miRNAs with 100% identity. We also used miRTrace^38^ to check the taxonomic identities of all the 683 miRNAs (Table S3).

### miRNA conservation analysis

To assess the conservation of novel miRNAs against known miRNAs, the mature sequence of each novel miRNA was input into BLASTn (v2.13.1+) to find similar sequences in the miRBase and FishmiRNA databases. The default *E*‐value threshold of 10 was used to report all hits.

### Quantification of miRNAs and differential expression analysis

To quantify the expression of each novel miRNA in all the samples, we used the quantifier.pl module in miRDeep2 and aligned the sequenced reads to the novel precursor miRNAs using the default parameters.

Differentially expressed miRNAs were determined using the R package edgeR^39^ (v3.22.3). The resulting *p*‐values from the differential expression analysis were adjusted using the Benjamini−Hochberg method with the R function p.adjust. miRNAs with an adjusted *p*‐value less than 0.05 and a fold change of at least two were considered as differentially expressed.

### mRNAseq dataset processing

Pachón and surface fish mRNA‐seq datasets for two time points, 24 hpf and 36 hpf, were downloaded from the SRA database: SRP045680. The reads were aligned to the *A. mexicanus* 2.0 reference genome, NCBI assembly GCF_000372685.2, using STAR^40^ (v2.7.3a). The gene model retrieved from Ensembl (release 102) was used to generate gene read counts. The transcript abundance TPM (transcript per million) was quantified using RSEM^41^ (v1.3). Differentially expressed genes were determined using the R package edgeR^39^ (v3.38.4). Prior to differential expression analysis, low‐expression genes were filtered out based on a cutoff of 0.5 CPM (counts per million) in at least one library. The resulting *p*‐values were adjusted with the Benjamini−Hochberg method using the R function p.adjust. Genes with an adjusted *p*‐value < 0.05 and a fold change of two were considered as differentially expressed.

### miRNA–mRNA target identification

miRNA–mRNA targets for the list of cave‐specific miRNAs were identified against the 3′ UTRs of all differentially expressed genes from the 24‐hpf and 36‐hpf time points using two different methods: miRanda^42^ (v3.3a) and TargetScan^43^ (v6.0).

For miRanda, targets were filtered using a score threshold of >80 and an energy threshold of < −14 kcal/mol. No additional filtering was applied when using TargetScan. The common targets identified by both miRanda and TargetScan were considered as the final miRNA–mRNA targets for each miRNA.

### GO analysis

Gene functional enrichment analysis or GO analysis was performed using a custom script built over the R package clusterProfiler^44^ (v4.4.4). *A. mexicanus* Gene–GO terms retrieved from Ensembl BioMart were used to identify overrepresented GO terms in the differentially expressed genes compared to the background list of all genes.

## RESULTS

### Identification and annotation of miRNAs in *A. mexicanus*

To establish a comprehensive repository of miRNAs in *A. mexicanus*, we sequenced, identified, and annotated small miRNA populations from surface fish and three distinct cavefish populations—Pachón, Tinaja, and Molino. Concurrently, we aimed to investigate the potential impact of miRNAs on the development of cave traits. One of the most obvious developmental differences between cave and surface fish occurs around 24 hpf, when the eye in cavefish starts to regress.^9^, ^45^, ^46^ Thus, we focused on 24 hpf embryos to explore differentially expressed miRNAs between cavefish and surface fish.

**FIGURE 1:**
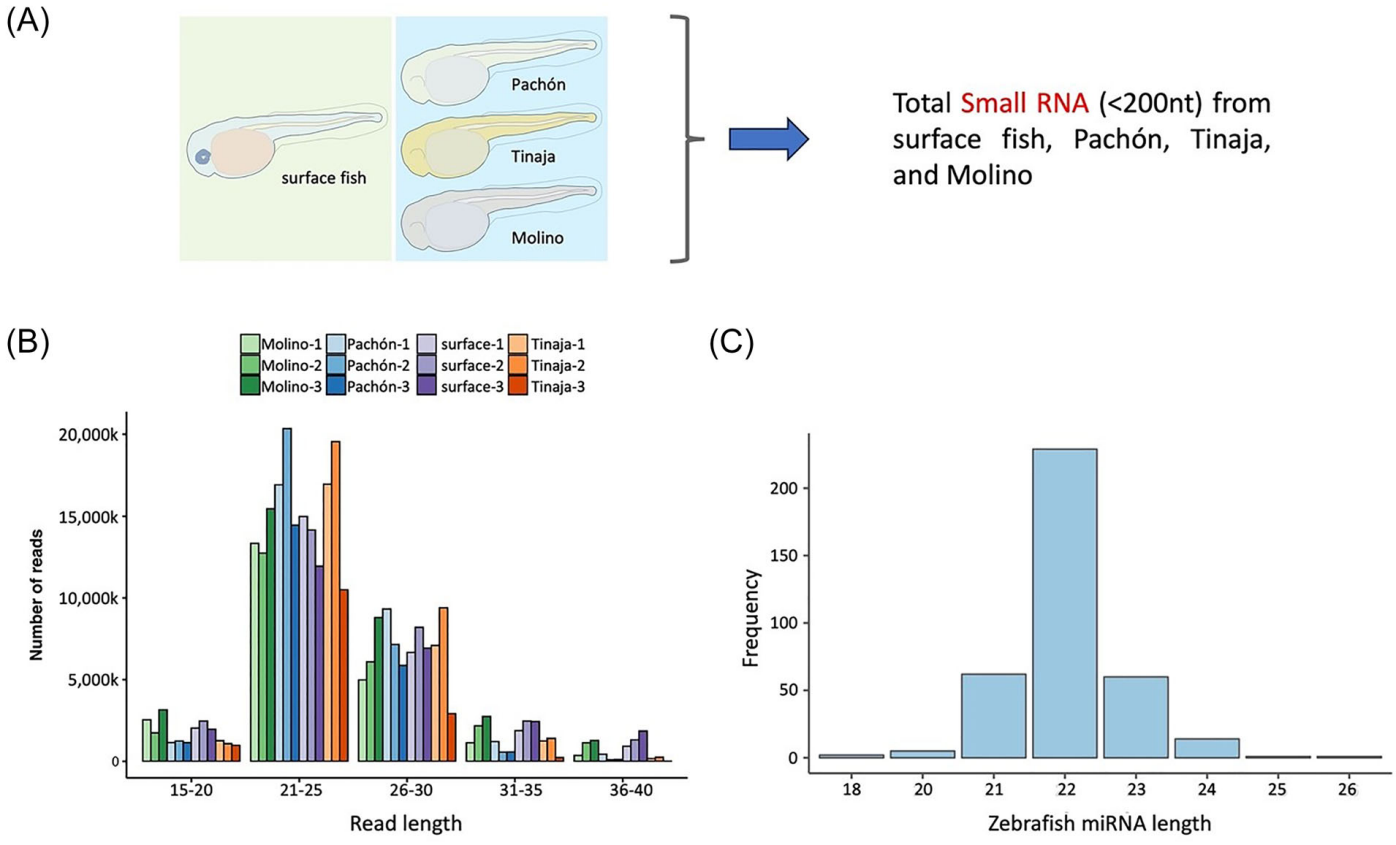
Identification of miRNAs in *Astyanax mexicanus*. (A) Graphical representation of the four morphs—surface fish, Pachón, Tinaja, and Molino at 24 hpf, from which the small RNA (<200 nt) population was collected. (B) Distribution of small RNA reads across read lengths of 15–40 bp. (C) Size distribution of all the zebrafish miRNAs available on miRBase.

To enhance the efficiency of miRNA identification, we specifically isolated and sequenced only small RNAs (<200 nt) instead of total RNA from the embryos (Figure 1A). After adapter trimming, the small RNA population with read lengths of 15–40 bp was found to be mostly enriched for reads in the range of 21–30 bp (Figure 1B). This was in line with the known range of miRNA lengths in zebrafish (Figure 1C). Subsequently, the reads were aligned to the *A. mexicanus* 2.0 genome and the trimmed files of all the samples were merged to call for novel miRNAs using miRDeep2^33^ with default parameters and zebrafish miRNA from miRBase as the supporting miRNA from related species. After removing redundancies using established pipelines (see Methods section), we identified and annotated a refined list of 683 mature miRNAs (Table S4) from an initial collection of 1452. We have also retained the genomic locations of origin of these mature miRNAs (Table S5). Of these 683 mature miRNAs, 681 were conserved across different organisms and had a hit in the miRBase^31^, ^32^ (Figure S2). Additionally, we also mapped the alignment of the *Astyanax* miRNAs against the curated database of teleost miRNAs available in the FishmiRNA database.^30^ Similar to the miRBase comparisons, we observed an alignment of 677 *Astyanax* miRNAs across different fish species. A similar comparison was also conducted with the MirGeneDB 3.0^47^, ^48^, ^49^, ^50^ database (Figure S3).

### Differential and cave‐specific expression of mature miRNAs across four morphs

**FIGURE 2:**
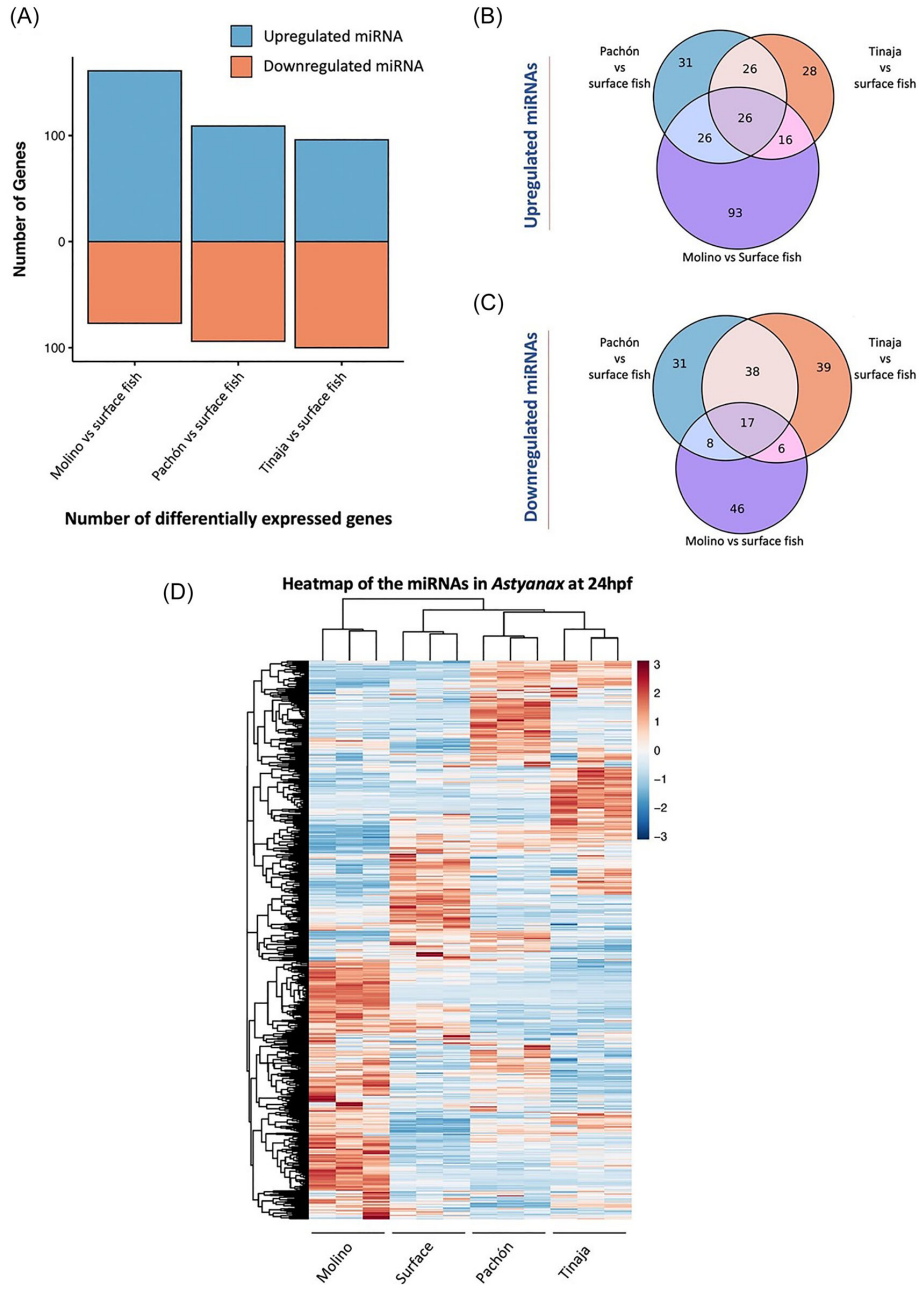
Differentially expressed mature miRNAs in cave morphs when compared to surface fish. (A) Abundance of differentially expressed miRNAs across Molino, Pachón, and Tinaja (arranged in order of abundance). (B) Venn diagram depicting the extent of overlaps in miRNAs upregulated in each of the cave morphs compared to surface fish. The central overlap of 26 miRNAs represents the cave‐specific miRNAs, upregulated in all the three cave morphs with respect to surface fish. (C) A similar Venn diagram as (B), depicting the extent of overlaps in miRNAs downregulated in each of the cave morphs compared to surface fish. (D) Heatmap based on the normalized counts of all 683 miRNAs expressed at 24 hpf across all the four morphs of *Astyanax*.

Small noncoding miRNAs are recognized for their role in facilitating evolutionary adaptation in organisms to environmental stresses.^51^, ^52^, ^53^ To explore any such correlation in cavefish development and adaptation, we investigated mature miRNAs that were differentially expressed in the cave morphs when compared to the surface fish. Comparing the miRNA expression profile at 24 hpf of each of the cave morphs (Pachón, Tinaja, and Molino) with that of the surface fish revealed both up‐ and downregulated miRNAs (Figure 2A). Pachón and Tinaja had similar numbers of differentially expressed miRNAs, totaling 203 and 196, respectively, while Molino exhibited the highest abundance of differentially expressed miRNAs among the three cave morphs, totaling 238. The highest number of upregulated miRNAs was observed in Molino, followed by Pachón and Tinaja, in comparison to surface fish. Conversely, the highest number of downregulated miRNAs was observed in Tinaja, followed by Pachón and Molino.

Next, we explored not just morph‐specific, but also cave‐specific differentially expressed mature miRNAs. We defined cave‐specific miRNAs as those miRNAs that are up‐ or downregulated across all the three cave morphs when compared to surface fish. We argue that cave‐specific miRNAs would be strong candidates for underlying early adaptation in cave morphs. From the list of all the differentially expressed miRNAs, 26 miRNAs were upregulated (Figure 2B) and 17 were downregulated (Figure 2C) across Pachón, Tinaja, and Molino, when compared to surface fish. The set of 26 and 17 miRNAs represents the first identified cave‐specific, noncoding miRNAs differentially expressed across three different cave morphs, with respect to surface fish. A comprehensive overview of the expression profiles across the different *Astyanax* morphs is provided by the heatmap, which is based on normalized counts for all 683 miRNAs (Figure 2D).

### Putative 3′ UTR targets of cave‐specific mature miRNAs in *A. mexicanus*

**FIGURE 3:**
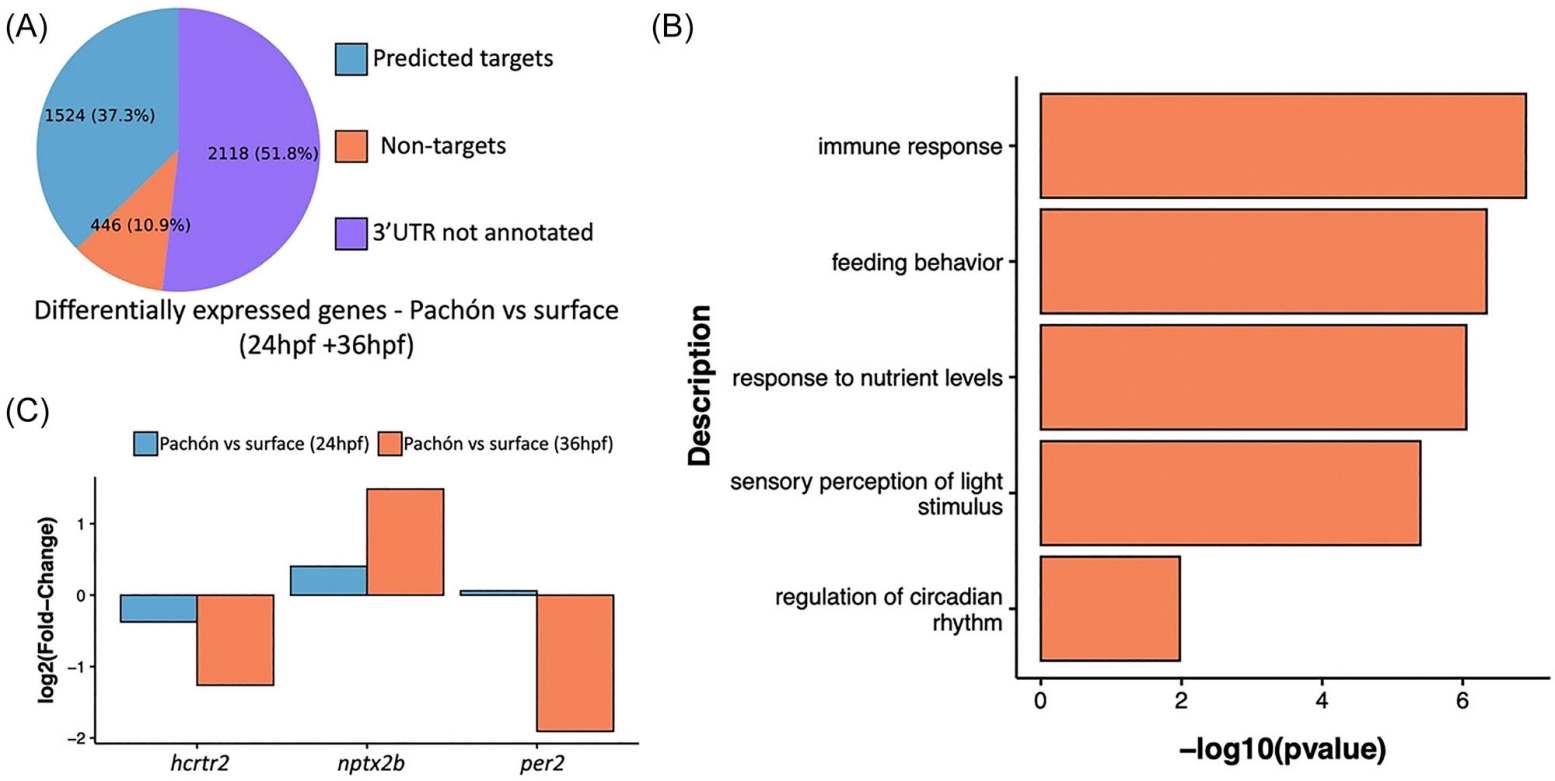
Differential expression of predicted targets of cave‐specific miRNA. (A) Pie‐chart depicting the percentage of differentially expressed genes that are also predicted to be targets of cave‐specific miRNA when Pachón is compared to surface fish at 24 hpf and 36 hpf. (B) Selected pathways from GO term analysis of all predicted and differentially expressed genes (1524) representing biological processes associated with cave adaptation. (C) Fold change of predicted targets *per2*, *hcrtr2*, and *nptx2b* at 24 hpf and 36 hpf.^54^

To further understand any possible functional implications of these cave‐specific miRNAs, we turned our attention to predicting their putative targets. miRNAs mostly target the 3′ UTR region of genes. To predict putative cave‐specific miRNA target genes in *A. mexicanus*, we used the established miRanda^42^ and TargetScan^43^ pipelines. It is to be noted that in *A. mexicanus*, only less than half of the annotated genes have an annotated 3′ UTR, specifically, 11,318 genes out of a total of 27,420 genes. The annotated 3′ UTRs span a considerable range of lengths, with a mean of around 2 kb (Figure S4). Therefore, in this study, we restricted our predictive pipelines to the list of genes with annotated 3′ UTRs and among the genes that were differentially expressed in an available sample set of mRNAseq data between Pachón—as a representative of cavefish—and surface fish as a control.^54^ From this dataset, we merged differentially expressed genes from Pachón versus surface fish at two time points, 24 hpf and 36 hpf. Our approach considered the time‐dependent nature of miRNA action, wherein it takes a few hours for the miRNA to be expressed at a certain time point to exhibit its effect.^55^, ^56^ Merging the two data points, we found 4088 unique differentially expressed genes in Pachón when compared to surface fish. Out of these differentially expressed genes, a total of 1524 genes were putative targets of the cave‐specific miRNAs as predicted by both the miRanda and TargetScan pipelines (Figure 3A, Tables S6 and S7), amounting to 37.37% of the differentially expressed genes. These 1524 genes are a collection of both the upregulated (Table S6) and downregulated (Table S7) cave‐specific miRNA's putative targets. Subsequently, we employed GO term analysis of these 1524 putative target genes to explore the biological pathways associated with them. Interestingly, several of the essential pathways commonly associated with cave adaptation were present in the list (Figure 3B). For example, we observed the enrichment of pathways associated with the regulation of circadian rhythm, as indicated the presence of *per2*, *hcrtr2*, and *nptx2b* in the list of differentially expressed genes that were also putative targets of miRNA (Figure 3C).^4^, ^57^, ^58^ We also observed an enrichment of neural pathways and pathways associated with immune systems. While cavefish neural projections and activity differ considerably from that of surface fish, owing to their modified sensory input systems, the immune system of cavefish is also different from that of surface fish, where cavefish exhibit decreased innate immune response compared to surface fish.^6^ Notably, even in an in vitro set‐up, an immune response has been observed to be one of the differentially regulated pathways between surface fish and cavefish cells.^59^

## DISCUSSION

In this study, we present, for the first time, a comprehensive catalog of 683 small noncoding mature miRNAs for the fish, *A. mexicanus*. Focusing on an early stage of development, we extracted and sequenced miRNAs from 24 hpf embryos of four different morphs of *A. mexicanus*—one being the extant surface fish, and the others were cave morphs of Pachón, Tinaja, and Molino. Analyzing the small RNAseq data, we found that each of the cave morphs exhibited more than 100 differentially expressed miRNAs compared to surface fish. The presence of differentially expressed miRNAs at the 24‐hpf stage suggests that not only are the miRNA‐mediated silencing pathways activated at an early stage in *A. mexicanus*, but are also potentially employed by cavefish morphs to facilitate cave adaptation. This idea is further strengthened by the fact that among the hundreds of differentially expressed mature miRNAs, there exists a set of mature miRNAs that were common across all the three cave morphs when compared to surface fish, making them essentially a cohort of cave‐specific miRNAs. We investigated further and performed in silico analysis to ascertain putative target genes from a list of differentially expressed genes at the relevant time points for each of the cave‐specific miRNAs. It was interesting to find a number of biological pathways associated with troglomorphic adaptation enriched when we performed GO term analysis on the list of putative targets of cave‐specific miRNAs. In the future, with the availability of better genomes^60^ for each of the morphs, it would also be interesting to incorporate not only more cave‐morphs of *Astyanax* into such small‐RNA studies but also other cavefish species to establish the relevance of the cave‐specific and morph‐specific role of miRNAs in development and evolution. Such comparative studies across multiple species with known genomes would be considerably aided by miRNA predictive tools such as MirMachine.^61^

While the target predictions have to be essentially validated functionally for a better understanding of the mechanistic role of the differentially expressed miRNAs in surface and cavefish, our present analysis suggests numerous developmental and metabolic adaptations essential for survival in cave environments that could begin at the early stages of development. Especially important for future functional analysis would be the miRNAs whose potential in silico targets are involved in the process of eye degeneration, circadian regulation, and oxidative pathways.

Additionally, there is another unique observation from the dataset that shows that the most numerous differentially expressed mature miRNAs were found in the Molino morph. Molino is one of the “younger” morphs that colonized the caves relatively recently, approximately 110,000 years ago.^2^ Molino lacks many of the known underlying alterations associated with cave evolution that are shared by the other two cave morphs of Pachón and Tinaja.^7^, ^8^ This observation hints at a possible dependence on miRNAs in Molino as a dynamic “rheostat” for fine‐tuning gene networks while adapting to a relatively new and extreme environment devoid of light and nutrients. Additionally, the miRNAs that are morph‐specific could also contribute to lineage specificity in *Astyanax* cave adaption. Both hypotheses, while exciting, need further functional testing.

Taken together, future studies into miRNAs, through gain‐ and loss‐of‐functions and target validation, hold a great potential to unravel new paradigms of gene regulation, development, as well as metabolism in cavefish. Our comprehensive catalog of miRNAs in *A. mexicanus* will, therefore, pave the way for exploring this hitherto unexplored facet of miRNA‐mediated adaptation and evolution of adaptation to new environments.

## AUTHOR CONTRIBUTIONS

T.B.: Conceptualization; investigation; analysis; visualization; original draft preparation. H.H.: Analysis; visualization; data curation. N.R.: Conceptualization; original draft preparation; funding acquisition.

## COMPETING INTERESTS

The authors declare no conflict of interest.

### PEER REVIEW

The peer review history for this article is available at: https://publons.com/publon/10.1111/nyas.15300

## Supporting information




**Figure S1**: Histogram showing the total range of sequencing reads in each sample.
**Figure S2**: Number of *A. mexicanus* miRNAs conserved across miRNAs of different species after miRBase alignment (zebrafish underlined in red).
**Figure S3**: Number of miRNAs which had a hit in miRBase, FishmiRNA, and in the MirGeneDB database along with number of miRNAs which did not have a hit.
**Figure S4**: Distribution of the 3′ UTR lengths of the 11,318 genes with annotated 3′ UTR in *Astyanax mexicanus*.

Supporting Information

Supporting Information

Supporting Information

Supporting Information

Supporting Information

Supporting Information

Supporting Information

## Data Availability

The RNA‐seq datasets have been uploaded to the GEO database with accession number GSE254598 (https://www.ncbi.nlm.nih.gov/geo/query/acc.cgi?acc=GSE254598). Original data underlying this manuscript can be accessed from the Stowers Original Data Repository at http://www.stowers.org/research/publications/libpb‐2454
