# Supplementary material for: Differentially expressed miRNAs offer new perspective into cave adaptation of Astyanax mexicanus
Source: Ann N Y Acad Sci. 2025 Mar 13;1546(1):173–81. doi: 10.1111/nyas.15300 (PMC11998478; doi:10.1111/nyas.15300)
Supplement: Supplementary file 1 — Figure S1: Histogram showing the total range of sequencing reads in each sample. Figure S2: Number of A. mexicanus miRNAs conserved across miRNAs of different species after miRBase alignment (zebrafish underlined in red). Figure S3: Number of miRNAs which had a hit in miRBase, FishmiRNA, and in the MirGeneDB database along with number of miRNAs which did not have a hit. Figure S4: Distribution of the 3′ UTR lengths of the 11,318 genes with annotated 3′ UTR in Astyanax mexicanus. [file NYAS-1546-173-s004.docx]

Supporting Figures


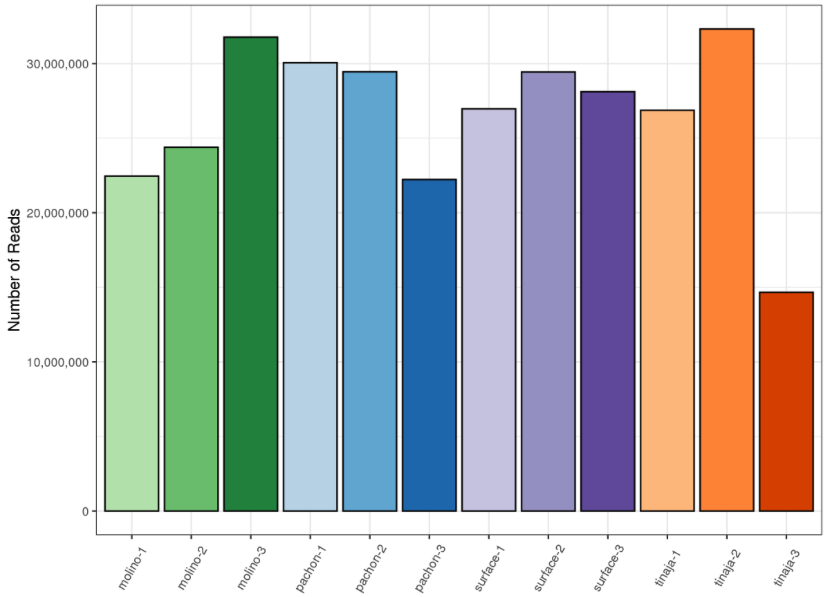


**Figure S1:** Histogram showing the total range of sequencing reads in each sample.

**
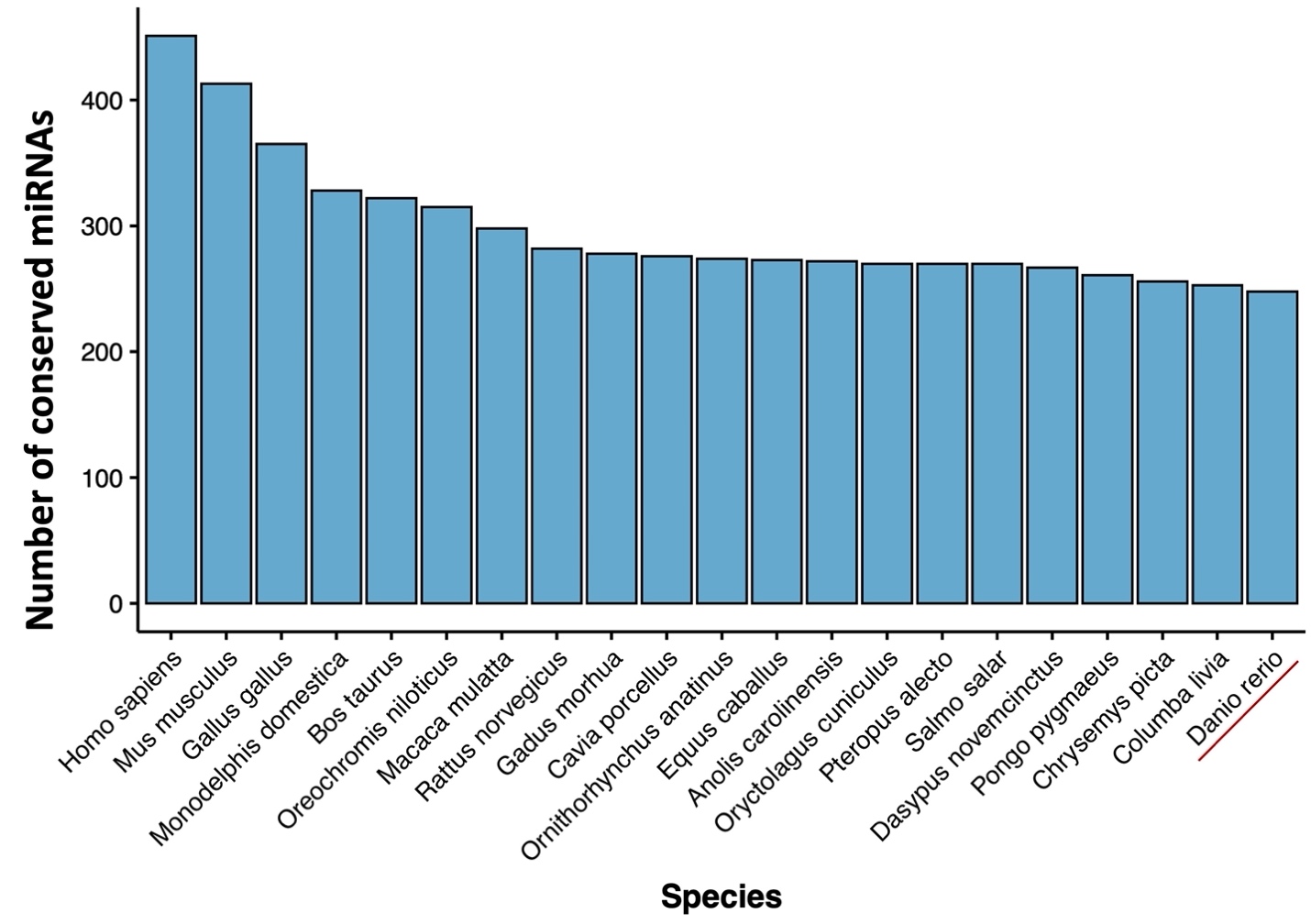
**

**Figure S2:** Number of *A. mexicanus* miRNAs conserved across miRNAs of different species after miRBase alignment (zebrafish underlined in red).


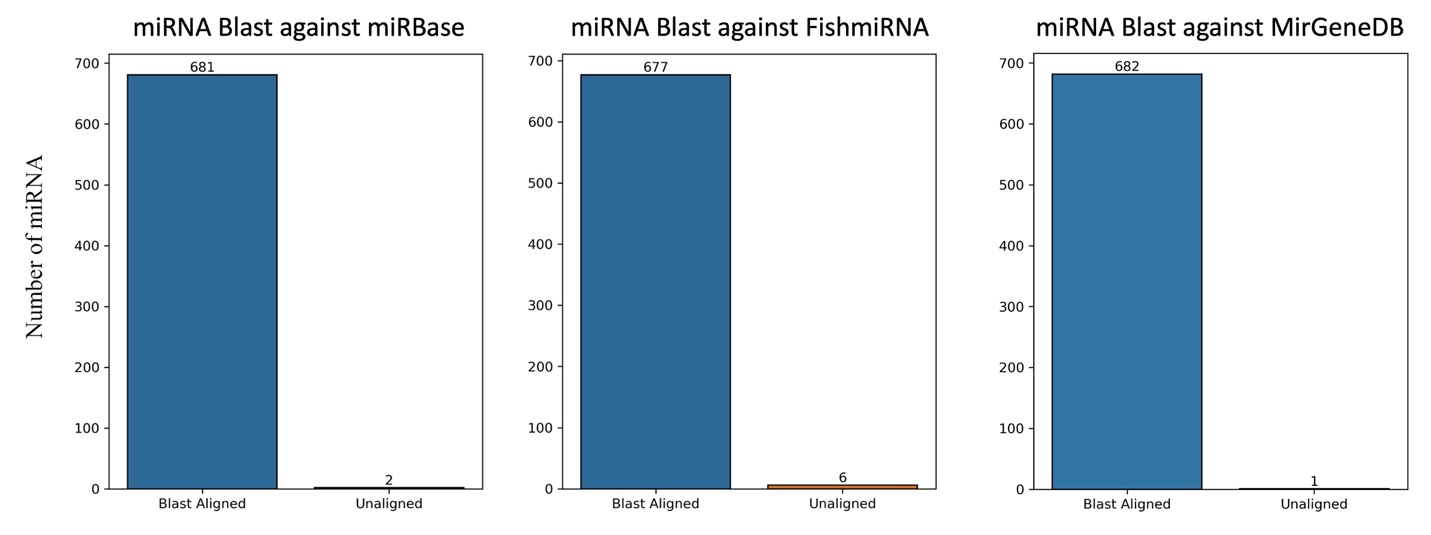


**Figure S3:** Number of miRNAs which had a hit in miRBase, FishmiRNA and in the MirGeneDB database along with number of miRNAs which did not have a hit.


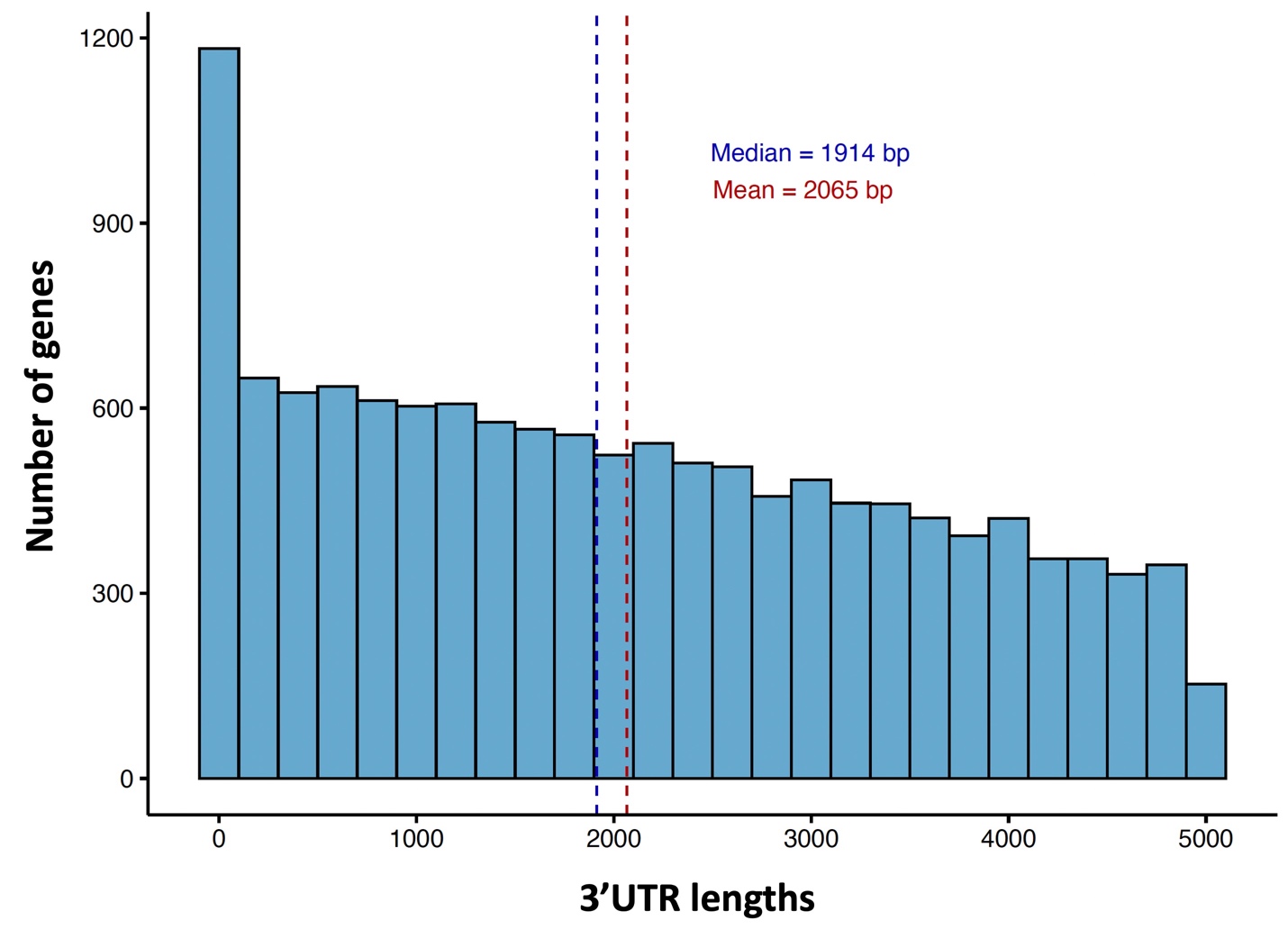


**Figure S4:** Distribution of the 3’UTR lengths of the 11,318 genes with annotated 3’UTR in *Astyanax mexicanus*.
